# Influence of first calving date on stayability and productivity in *Bos indicus-Bos taurus* crossbred cows

**DOI:** 10.1093/tas/txag051

**Published:** 2026-04-28

**Authors:** Bailey N Engle, Clare A Gill, Jason E Sawyer, David G Riley, James O Sanders, Andy D Herring

**Affiliations:** USDA, ARS, U.S. Meat Animal Research Center, Clay Center, NE, 68933, United States; Department of Animal Science, Texas A&M University, College Station, TX, 77843-2471, United States; Department of Animal Science, Texas A&M University, College Station, TX, 77843-2471, United States; Department of Animal Science, Texas A&M University, College Station, TX, 77843-2471, United States; East Foundation, San Antonio, TX, 78216, United States; Department of Animal Science, Texas A&M University, College Station, TX, 77843-2471, United States; Department of Animal Science, Texas A&M University, College Station, TX, 77843-2471, United States; Department of Animal Science, Texas A&M University, College Station, TX, 77843-2471, United States

**Keywords:** beef heifers, calf performance, cow productivity, first calving period, Nellore-Angus cross, stayability

## Abstract

Reproductive longevity is directly related to a cow’s lifetime productivity and cumulative economic value, making it one of the most important influences on herd profitability. Therefore, it would be advantageous to identify component traits correlated to longevity that can be measured and used as selection criteria in young replacement prospects. A herd of *Bos indicus–Bos taurus* crossbred cows (*n* = 293) were used to (i) characterize the relationship between first-parity calving season period with subsequent longevity and cow productivity and (ii) assess the influence of the cow’s birth period on her potential for stayability and productivity. Calving period was assigned by designating each of the calving seasons into four 21-d periods, and each cow was assigned a score of 1–4 for the time period of her first parturition and her own birth. The relationship between calving period and stayability to ages 3, 4, 5, 6, and 7 yr of age, first and lifetime average calving intervals, first-calf weight, lifetime average calf weights, and cumulative calf weight weaned were evaluated. There was a clear advantage for heifers calving within the first 21 d of the calving season as their calves were heavier at weaning (*P* < 0.05) and demonstrated greater preweaning weight gain (*P* < 0.05). This was further reflected in long-term productivity, as cows that calved in the first 21-d period of their first-parity calving season produced greater average calf crop weaned (*P* < 0.05) and greater cumulative calf crop weaned (*P* < 0.05) than cows that calved later in their first season. These early-calving heifers were more likely to meet stayability benchmarks at 3, 4, 5, 6, and 7 yr than heifers calving at later periods in the season (*P* < 0.05). Earlier born cows had a higher probability of heifer pregnancy (*P* < 0.05) and were heavier at first-calf weaning (*P* < 0.05), possibly because they were older at first calving (*P* < 0.05). Due to the advantages to both maternal productivity and calf performance, calving within the first 21 d of an annual calving season is expected to increase the herd longevity of these females, and may be considered as an earlier-in-life evaluation criterion when selecting for longevity or lifetime productivity in *B. indicus-B. taurus* crossbred beef cows.

## Teaser

Heifers calving within the first 21 d of their first calving season had advantages of maternal productivity, longevity, and calf performance.

## Introduction

Beef cow reproductive longevity is related to a cow’s lifetime productivity and cumulative economic value, making it one of the most important factors influencing herd profitability (Rogers 1972). However, Hudson and Van Vleck (1981) argued against direct selection for longevity due to low heritability, increased generation interval, and automatic selection via older cows that contribute more offspring to subsequent generations than do short-lived cows. Due to these arguments, it would be advantageous to identify component traits correlated to longevity that can be measured and applied as culling criteria earlier in a cow’s life.

It is anticipated that when heifers conceive earlier in their first breeding season, they will calve earlier in the subsequent calving season. Calving early as primiparous cows allows for more time for recovery postpartum, increasing the likelihood that the cow will return to estrus in time to rebreed during the following breeding season (Lamb et al. 2001; Diskin and Kenny 2014). Cows are then more likely to calve early in the following calving seasons, thus, repeating the cycle. Prior work has shown that *Bos taurus* heifers that calved in the first 21 d of their first calving season experienced increased longevity compared to heifers that calved later in their first calving season (Cushman et al. 2013; Damiran et al. 2018). This is expected to be at least as important in *Bos indicus-*influenced herds, as these heifers are known to be older at the onset of puberty than their *B. taurus* counterparts (Plasse et al. 1968; Thallman et al. 1999). Delayed puberty has a negative effect on a female’s ability to first calve at 2 yr of age, rebreed, and calve again at 3 yr, depressing their potential for long term productivity and life in the herd (Chenoweth 1994) within a typical U.S. production system. The timing of when a heifer gives birth during her first calving season is also predictive of the performance of her future progeny (Lesmeister et al. 1973; Funston et al. 2011; Mousel et al. 2012). These studies suggest that the relative calving date in the first-parity calving season has an influence on cow profitability in weight of calf weaned in *B. taurus* females. However, these patterns have not yet been assessed in *B. indicus* or *B. indicus*-influenced herds.

The primary objective of this study was to characterize the relationship between the first-parity calving period of *B. indicus-B. taurus* crossbred heifers with subsequent longevity and cow productivity. We hypothesized that relative timing of first-parity calving would be negatively correlated with reproductive longevity, where an earlier first-calving date increased the likelihood of greater longevity. The relationship between a cow’s own relative date of birth and her potential for stayability and cow productivity was also evaluated.

## Materials and methods

All procedures involving animals were approved by the Texas A&M University Institutional Animal Care and Use Committee.

### Population

Cows assessed in this study were part of the McGregor Genomics Cycle 1 Population (*n* = 293), an experimental herd housed in McGregor, Texas at the Texas A&M AgriLife Research Center. This population has previously been described by Engle et al. (2018). Briefly, these cows were all *B. indicus–B. taurus* crosses, specifically, Nellore–Angus × Brahman–Hereford crosses (*n* = 60), Nellore–Angus × Brahman–Angus crosses (*n* = 48), and Nellore–Angus F_2_ crosses (*n* = 185); all were 50% *B. indicus* and 50% British (*B. taurus*). Cows were from either 4 paternal half-sibling families produced through natural service or 13 full-sibling, embryo transfer F_2_ families (i.e., Nellore–Angus F_2_ crosses). Four Nellore-Angus F_1_ bulls sired all cows, and all sires are represented in each of the 3 breed-crosses. From 2003 through 2007, cows were born either during spring (*n* = 224) or fall (*n* = 69) calving seasons. Records used for this analysis span from the date of this population’s first possible calving season in 2005 through 2015, which is when the project ended for a portion of the population, and when the youngest cows in the herd were at least 8 yr of age.

All heifers were exposed to Angus bulls for the opportunity to first calve at approximately 2 yr of age. Fall-born heifers (*n* = 69) were exposed to bulls from the first week in December to the second week in February and given the opportunity to first calve at 2 yr of age in the following fall. Due to environmental challenges associated with fall breeding *B.* *indicus* heifers (Randel 1984), those that initially failed to conceive were transitioned to a spring calving schedule and were bred to first calve at 2.5 yr of age (*n* = 60), without a failure to calve counted against them. Any fall-born heifers that first calved during the fall (*n* = 9) were held through the subsequent winter without mating opportunity, and then rebred in the following spring breeding season to be on a spring calving schedule, with their second calf born at 3.5 yr of age. Spring-born heifers (*n* = 224) were all managed to first calve at 2 yr of age. Subsequently, all cows were managed together for spring calving only. Across the study, the average length of the breeding season was 68 d. Once a cow experienced two incidences of failure to wean a calf, under actual management criteria the cow was removed from production.

### Phenotypes and descriptive variables

**Figure 1:**
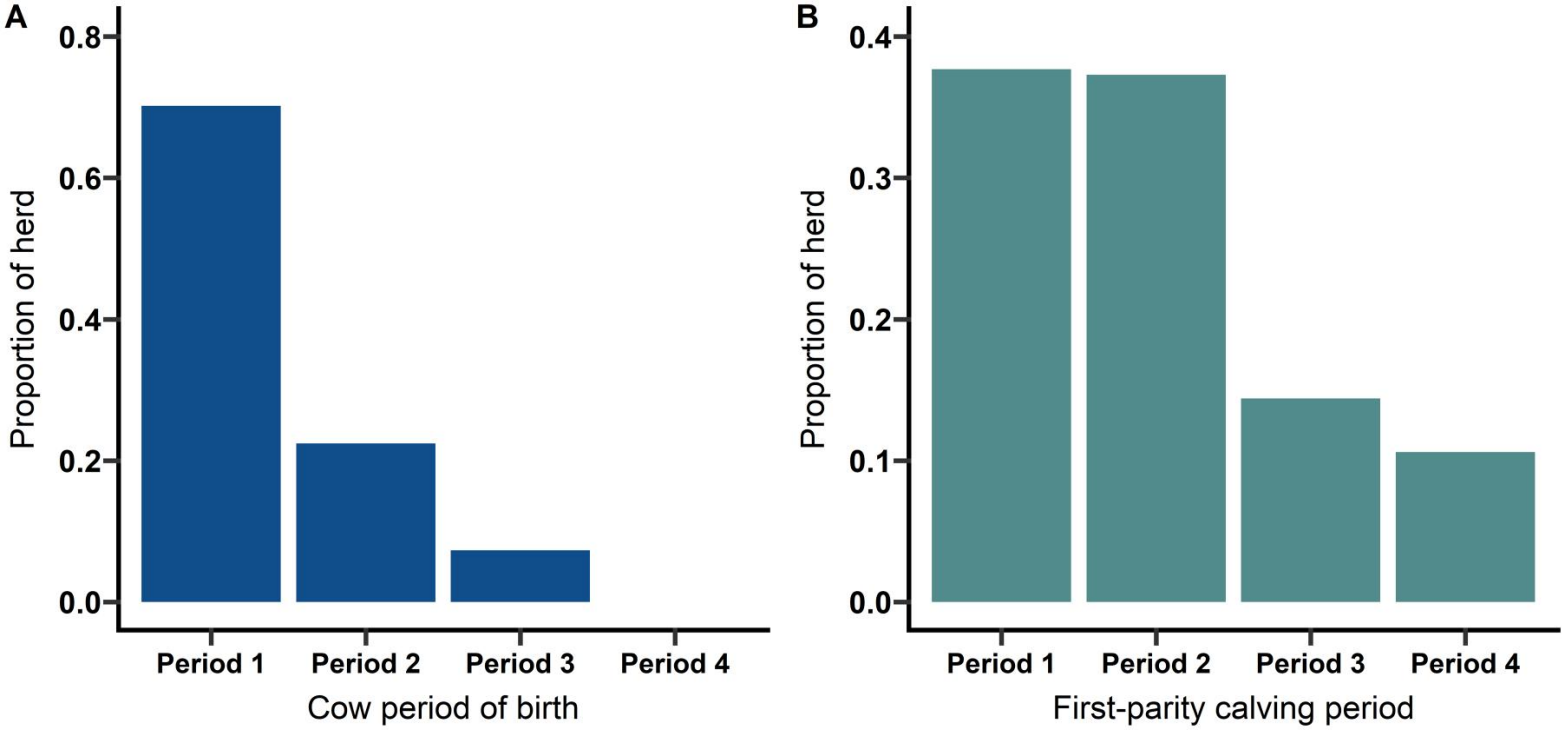
Observed proportions of (A) cows and their (B) first-parity calves born within each period of their respective calving seasons. Period 1: ≤ 21 d, period 2: 22 to 43 d, period 3: 44 to 63 d, period 4: ≥ 64 d.

Calving period was assigned by classifying each of the calving seasons into 21-d periods, reflecting the standard estrous length in cattle. The average length for the 5 calving seasons evaluated was 80 d, so each season was split into 4 levels and treated as a categorical variable. Although the beginning dates vary across years, Period 1 represented the first 21 d of the season, Period 2 spanned d 22–43, Period 3 equated to d 44 through d 63, and Period 4 included everything after d 64 (Fig. 1). The calving season in which the cow was born was categorized using the same 21-d scheme, and each cow was assigned a score of 1–3 for the period when she was born (Fig. 1). No cows in this study were born in Period 4 of their respective birth seasons.

Stayability has been defined as a cow’s probability of surviving to a specific age, given the opportunity to first reach that age (Hudson and Van Vleck 1981), and was used as a proxy for longevity. Stayability was evaluated to ages 3, 4, 5, 6, and 7 yr, provided that a cow calved each year starting at 2 yr of age (Snelling et al. 1995). Stayability was evaluated as a binary threshold trait where records were censored and cows were scored as either a 1, representing successfully reaching a given age, or a 0, representing calving failure at or before the given age. For each cow, production records were censored upon her first failure to give birth to a calf, as actual management practice at the research center allowed for two reproductive failures prior to culling. Due to the way that stayability was scored in these analyses, any individual that did not calve during their first calving season at approximately 2 yr of age would not have met any of the stayability benchmarks, and were therefore removed from stayability, heifer productivity, and cow productivity analyses. All cow records were used to assess the relationship between their own period of birth and probability of successful heifer calving, modeled as a binary trait.

First-parity calving interval was measured from the birth date of the first calf to the birth date of the second calf. Average calving interval over the cow’s lifetime was calculated as the mean difference in days between all calf birth dates. The unadjusted body weights at birth and weaning of the cow’s first calf were evaluated. Average and cumulative weight of calves weaned over the course of each cow’s productive life were evaluated up to 8 yr of age. Average calf birth weight across a cow’s productive life was also evaluated. Analyses of a cow’s lifetime average calf weights only included cows with more than one calf record. Cumulative productivity measures included all cows with weaning records, allowing for the advantage of multiple parities to be accounted for in the model (Snelling et al. 2019). Cow bodyweights were collected annually at calf weaning and averaged across the length of each cow’s productive life in the herd (2–8 yr). Eight years was the maximum age that every cow was maintained in the herd, prior to the project ending for a portion of the population due to drought conditions. Consequently, some production records beyond 8 yr were censored.

Possible fixed effects included the categorical effects of calving period, breed-cross, cow’s sire, and concatenated cow birth year-season, calf sex, and covariates for cow bodyweight at first-calf weaning, lifetime cow average bodyweight, and cow age in days at first calving. Prior to analyses, all continuous traits, including both response and independent variables, were tested for normality using a Shapiro-Wilks test, and outliers were removed if they exceeded 1.5 times the interquartile range.

### Modeling and variable selection

To assess binomial stayability to each age, a generalized linear model with a probit link was utilized, using the *glm* function in R (version 4.5.1) (R Core Team 2025). The model used for the analysis was: $f(\mu_{Y})=X\beta +\varepsilon$, where $f(\mu_{Y})={Ф}^{-1}(\mu_{Y})$ and is the inverse normal distribution of the binomial response variable Y, X is the explanatory variable, β is the coefficient of change for the explanatory variable, and ε is the model error term.

Prior to analysis, all possible covariates were tested for model significance using a stepwise variable selection process. Final stayability models included the fixed effects of categorical calving period and cow weight at calf weaning, and models for binary heifer traits also included cow birth year-season. Cow age at first calving was not significant in any model, likely because the largest differences (i.e.; first calving at 24 mo vs. 30 mo) were almost entirely confounded with contemporary group (birth year-season).

To assess calving interval, calf birth weight, and cow productivity in the form of calf weight weaned, linear modeling was performed using the *lm* function in R (version 4.5.1) (R Core Team 2025). Prior to analysis, all possible covariates were tested for model significance using a stepwise variable selection approach. Statistical analysis of each productivity trait fit an individualized, model-specific combination of significant explanatory variables, with each model including the fixed effect of categorical calving period. The main effect and significance of fixed effects were estimated for each model using *anova* in R (version 4.5.1).

Least-squares means for calving periods in each of the previously described analyses were estimated using the *lsmeans* package in R (version 2.26–3; Lenth 2016) and back transformed from probit to response scale to aid in interpretation. Pairwise contrasts between calving period levels were tested using a Tukey pairwise comparison on the probit scale, and significance was declared if *P* < 0.05.

Additional tests were performed to assess the influence of the available fixed effects on calving period. In modeling both the cow’s relative birth period and her first-parity calving period, day of birth within a season were fit as continuous response variables within linear models instead of the categorical classification. Least-squares means were estimated for categorical fixed effects and compared using the previously described procedure.

## Results

The primary objectives of this study were to characterize the relationship between the first-parity calving period of *B. indicus-B. taurus* crossbred heifers with subsequent longevity and productivity. Heifers that calved within the first 21-d calving period were the most likely to rebreed and calve again at 3 yr of age, whereas heifers that calved in the fourth calving period were less likely to rebreed (*P* < 0.05; Table 1). This pattern was again observed for stayability to 4 yr where cows that first calved within Period 1 were more likely to meet the stayability threshold (*P* < 0.05; Table 1). The benefit of first-parity heifers calving in Period 1 of their first calving season became more pronounced as stayability was assessed to later ages, where heifers that calved in Period 1 were significantly more likely to meet stayability benchmarks to 5 and 7 yrs than heifers that calved within any other calving period (*P* < 0.05; Table 1).

**Table 1:** Least squares means for probability of meeting stayability benchmarks relative to first-parity calving period.

	First-parity calving period^1^				Observed frequency of success^3^
	1	2	3	4	
**Heifers (n)**	89	88	34	25	
**Stayability** ^2^ **: 3 yr**	0.92^a^ ± 0.03	0.80^ab^ ± 0.04	0.63^bc^ ± 0.08	0.50^c^ ± 0.10	0.67
**Stayability** ^2^ **: 4 yr**	0.82^a^ ± 0.04	0.70^ab^ ± 0.05	0.60^ab^ ± 0.08	0.50^b^ ± 0.10	0.60
**Stayability** ^2^ **: 5 yr**	0.83^a^ ± 0.04	0.65^b^ ± 0.05	0.52^b^ ± 0.09	0.38^b^ ± 0.10	0.56
**Stayability** ^2^ **: 6 yr**	0.77^a^ ± 0.05	0.60^ab^ ± 0.05	0.49^b^ ± 0.09	0.33^b^ ± 0.10	0.52
**Stayability** ^2^ **: 7 yr**	0.73^a^ ± 0.05	0.53^b^ ± 0.05	0.46^b^ ± 0.09	0.33^b^ ± 0.10	0.47

1 The 21-d period during a heifer’s first-parity calving season, where Period 1: ≤ 21 d, Period 2: 22 to 43 d, Period 3: 44 to 63 d, Period 4: ≥ 64 d.

2 Indicates a binary trait, where a 1 = success and 0 = failure to meet each threshold, presented as a probability (back transformed least squares means to response scale from a probit link).

3 Proportion of cows from entire herd meeting each stayability benchmark, where whole herd *n* = 293.

a,b,c Indicates significant difference of at least *P* < 0.05, pairwise tests performed on probit scale.

Heifers in this study that calved in the first-calving period had the longest calving interval between their first and second calf (*P* < 0.05; Table 2). This represents a systematic benefit, allowing those heifers that calved early more time for recovery and return to estrus prior to the next breeding season. While the overall fixed effect of first-calving period on lifetime average calving interval accounted for a significant proportion of model variance (*P* = 0.03), no significant pairwise differences between calving periods were observed (*P* > 0.05; Table 2). However, the average length of calving intervals across a cow’s lifetime tended to increase with later first-calving periods, with differences between Periods 1 and 3 approaching statistical significance (*P* = 0.057; Table 2).

**Table 2:** Least squares means for cow productivity traits relative to first-parity calving period.

	First-parity calving period^1^			
	1	2	3	4
**Heifers (n)**	89	88	34	25
**1^st^ calving interval, d**	384.2^a^ ± 1.9	371.0^b^ ± 2.2	356.1^c^ ± 3.7	334.9^d^ ± 5.2
**Lifetime mean calving interval, d**	383.0^x^ ± 3.5	389.3 ± 3.8	399.9^y^ ± 5.8	397.9 ± 7.7
**1^st^ calf birth wt, kg**	27.8^a^ ± 0.4	29.7^b^ ± 0.5	30.6^b^ ± 0.7	30.5^b^ ± 0.9
**Mean calf birth wt, kg**	32.5 ± 0.4	32.2 ± 0.4	31.9 ± 0.6	31.2 ± 0.8
**1^st^ calf weaning wt, kg**	207.2^a^ ± 2.4	194.4^b^ ± 2.5	181.0^c^ ± 3.9	167.4^c^ ± 5.0
**Mean calf weaning wt, kg/cow**	209.7^a^ ± 2.0	202.8^ab^ ± 2.1	196.8^bc^ ± 3.6	184.9^c^ ± 4.2
**Cumulative calf wt weaned, kg/cow**	1138^a^ ± 54	940^ab^ ± 57	781^bc^ ± 88	608^c^ ± 104

1 The 21-d period during a heifer’s parity first calving season, where Period 1: ≤ 21 d, Period 2: 22 to 43 d, Period 3: 44 to 63 d, Period 4: ≥ 64 d.

a,b,c,d Indicates significant difference of at least *P* < 0.05.

x,y Indicates difference with *P* = 0.057.

A heifer’s first calving period had a significant effect on the performance of her first calf and lifetime productivity (Table 2). First-calf birth weights were lighter in early-born calves from Period 1 than calves born in all other calving periods (*P* < 0.05). There was no effect of first calving period on a cow’s lifetime average for calf birthweight (*P* = 0.30). First-calf weaning weight decreased by advancing periods (*P* < 0.05; Table 2), where the heaviest calves were born in Period 1 of the season (*P* < 0.05; Table 2). These early-in-life benefits appear to extend beyond a cow’s first calf, as cow lifetime average calf weaning weight decreased by later first-calving period (*P* < 0.05; Table 2). Heifers that calved in Period 1 of their first calving season produced 198 to 530 more kilograms of cumulative calf crop weaned than heifers calving in other periods (Table 2), reflecting a greater potential for lifetime productivity from early calving heifers.

The relationship between a cow’s own date of birth within a season and her potential for stayability and cow productivity were also evaluated. The calving period a cow was born in was not shown to influence their ability to successfully meet a stayability threshold at ages 3, 4, 5, 6, and 7 yr (*P* > 0.40). There was also no impact of a cow’s birth period on the weight of her first calf at either birth (*P* = 0.52) or weaning (*P* = 0.13), nor on a cow’s lifetime average for calf birth weight (*P* = 0.44) or calf weaning weight (*P* = 0.26). Unsurprisingly, there was not a significant effect of cow birth period on cumulative calf crop weaned (*P* = 0.33). There was, however, a significant relationship found between a cow’s birth period on her productivity as a heifer and cow size. Earlier born cows had an increased probability of successful heifer pregnancy (*P* < 0.05; Table 3); These cows were older at first calving (*P* < 0.05; Table 3), and therefore older at breeding. Earlier born cows also tended to be heavier at the weaning of their first calf (*P* < 0.05; Table 3). Although the calving interval between first and second calves was up to 9 d longer for cows born in Period 1 (*P* < 0.05; Table 3), there was no period effect on likelihood of heifer rebreeding (*P* = 0.44). Finally, while the overall model effect of a cow’s period of birth on mature cow size was significant (*P* = 0.01), no pairwise differences between periods were estimated (*P* > 0.09, Table 3).

**Table 3:** Least squares means for cow productivity traits relative to her period of birth.

	Cow period of birth^1^		
	1	2	3
**Cows (n)**	172	55	18
**Age at first calving, d**	803.7^a^ ± 3.2	796.8^ab^ ± 4.6	780.4^b^ ± 7.5
**1^st^ calving interval, d**	376.6^a^ ± 2.1	365.7^b^ ± 3.7	367.4^ab^ ± 6.3
**Cow weight at 1^st^ calf weaning, kg**	411.2^a^ ± 3.1	403.6^ab^ ± 5.3	389.0^b^ ± 8.9
**Mean mature cow weight, kg**	479.5 ± 5.5	467.2 ± 7.4	453.9 ± 12.3
**Cows (n)**	197	70	26
**Heifer pregnancy success^2^**	0.96^a^ ± 0.02	0.92^ab^ ± 0.04	0.77^b^ ± 0.10

1 The 21-d period during a calving season when a cow was born, where Period 1: ≤ 21 d, Period 2: 22 to 43 d, Period 3: 44 to 63 d, Period 4: ≥ 64 d.

2 Binary trait, where a 1 = success and 0 = failure. Least squares means presented as a probability, back transformed to response scale from a probit link. Pairwise tests performed on probit scale.

a,b Indicates significant difference of at least *P* < 0.05.

**Figure 2:**
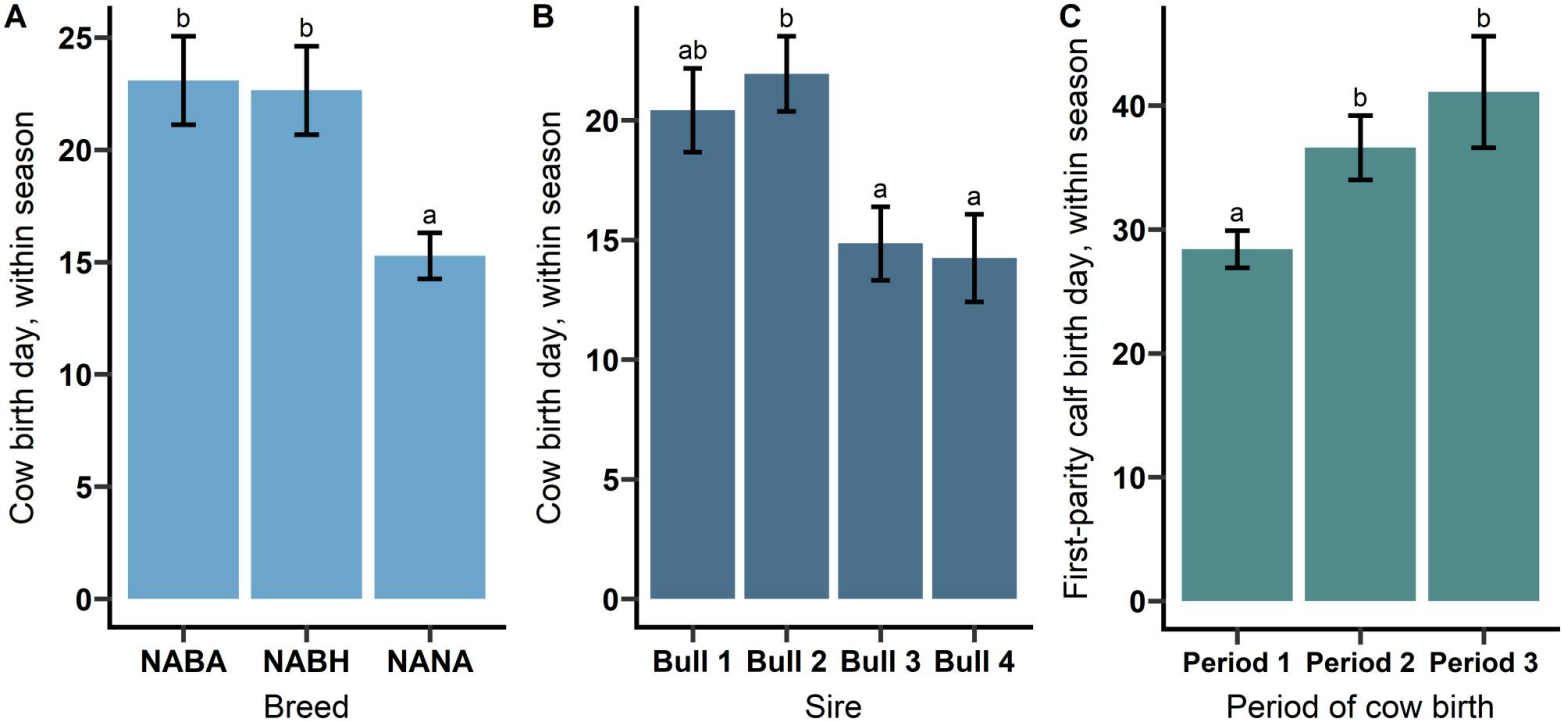
The relationship between (A) breed, (B) sire, and (C) dam period of birth on the relative day within a calving season that an animal is born. Different letters indicate statistically significant differences (*P* < 0.05). differences between bull 1 vs. Bull 3 and bull 1 vs. Bull 4 was *P* ≤ 0.081. Cow breeds are Nellore–Angus × Brahman–Angus crosses (NABA), Nellore–Angus × Brahman–Hereford crosses (NABH), and Nellore–Angus F_2_ crosses (NANA). Period 1: ≤ 21 d, period 2: 22 to 43 d, period 3: 44 to 63 d.

Birth date within annual calving season was found to be associated with multiple tested factors. Cows in this project represent 3 breed-cross combinations and were all sired by 4 bulls, who sired cows within each breed-cross grouping. The relationship between cow breed-cross and sire of cow with relative date of birth within a season were tested by transforming the categories of calving period (1–4) into a continuous variable of day within the season. The day that each cow was born within a season (1–61 d) was significantly influenced by sire (*P* = 0.001) and breed-cross (*P* < 0.001), with significant differences between sires and breeds (*P* < 0.05; Fig. 2). All Nellore-Angus F_2_ crosses were produced from embryo transfer, whereas the other two crosses were produced from natural service, and the confounding effects of breed and management cannot be separated in this dataset. Regardless, there still appears to be a genetic effect on the calving date in which a heifer is born, and this has desirable management and potential genetic selection advantages. The relative day within a season that a heifer’s first calf was born was not associated with the dam’s breed-cross or the dam’s sire (*P* > 0.20), but was influenced by the calving period that the dam was born in. Heifers that were born in Period 1 of their own birth season first calved 8.2 d earlier than heifers born in Periods 2 or 3 (*P* < 0.05; Fig. 2).

## Discussion

These results suggest that in a *B. indicus-B. taurus* crossbred population, the birth date of a heifer’s first progeny has a significant impact not only on the performance of that calf, but also on her long-term potential for cow productivity. We hypothesized that the relative date of heifer’s first parturition would be negatively correlated with reproductive longevity, where an earlier first-calving date increased the likelihood of greater longevity, and these results support this hypothesis. Early-calving heifers were more likely to meet stayability benchmarks at 3, 4, 5, 6, and 7 yr than heifers calving later in the season. This is comparable to previous studies that found *B. taurus* heifers that calved earlier during their first calving season, or the first 21-d period of the season, had an increased productive lifespan and herd retention in comparison to those that first calved in later periods (Mousel et al. 2012; Cushman et al. 2013; Damiran et al. 2018).

This and other studies highlight the implied economic advantages of first parity occurring within the first 21 d of the calving season as heifers’ first calves are heavier at weaning and demonstrate an increased preweaning weight gain (Funston et al. 2011). The date of first parturition for *B. taurus* first-calf heifers influences the weaning weights of not only their first calf, but also the weaning weights of their subsequent calves (Lesmeister et al. 1973; Arthur et al. 1993; Funston et al. 2011; Mousel et al. 2012), and our study supports this same effect in *B. indicus-B. taurus* crosses. Calves born earlier in the season will be older at weaning and are therefore more likely to be heavier at weaning (Lesmeister et al. 1973; Arthur et al. 1993). Due to the advantages to both maternal productivity and calf performance, calving within the first 21 d of a calving season is expected to increase the herd longevity of these females, providing opportunities to wean more, and heavier, calves.

This study showed an advantage for heifers calving in the first 21-d period of the calving season on heifer rebreeding rates, likely the luxury of a longer calving interval following first parturition (Lamb et al. 2001; Diskin and Kenny 2014). Lamb et al. (2001) found that pregnancy rates were 11% lower in cows that were less than 50 days postpartum at the beginning of the breeding season than cows that had more time to recover, highlighting the benefits of early calving. An improvement in future cow fertility for these heifers was also observed, reflected by a greater probability for calving success and lifetime of shorter calving intervals for early calving heifers. Calves born earlier in annual calving seasons will be older and likely heavier at weaning (Cushman et al. 2013), an advantage that was found to be maintained over the cow’s productive lifetime. The combined effect of these factors is reflected by the cow’s cumulative weight of calves weaned, where cows that produced more and heavier calves were at an advantage (Snelling et al. 2019). Unsurprisingly, when heifers calved in Period 1 of their first-parity calving season they recorded larger totals for cumulative calf crop weaned, representing an overall, lifetime productivity advantage for early calving heifers.

It has been previously reported that the calving period a breeding female was born in can have an impact on the performance of her offspring (Funston et al. 2011). An early-born heifer’s first calf was lighter at birth and heavier at weaning than progeny of later born, first-calf heifers (Funston et al. 2011). These patterns were not directly observed in this study. Given that calf birth period was associated with multiple weight outcomes, it is likely that this study does not have enough power to fully estimate the relationship between cow birth period and calf performance. Here, 63% of cows were produced using embryo transfer, and there was a more constricted calving season for their births than the natural-service sired females. Of the cows born from embryo transfer, 37% were fall born, and 87% of fall-born heifers first calved at 2.5 yr of age. Alternatively, all spring-born heifers first calved closer to 2 yr. While these effects were accounted for in the analyses, the unbalanced data structure and relatively low animals numbers likely reduced the number of significant associations estimated between cow birth period and calf performance. However, a dam’s period of birth was found to influence the period of birth her first calf would be born in, where earlier born heifers calved earlier in their first season. This suggests that from a management timing perspective, earlier born cows may positively influence their future daughters’ potential for breeding success.

Funston et al. (2011) also reported additional benefits of an early calving date in the form of improved heifer body weight at both pre-breeding and pre-calving, greater percent pre-breeding cycling, and as a result, greater pregnancy rates than those heifers born later in the season. Although no puberty phenotypes were directly collected in this population, similar trends for increased heifer pregnancy rates were observed. Heifer body weight at calf weaning was available for this study and was desirably associated with first calving date, potentially reflecting similar differences in heifer maturity and advantages for heifer fertility.

The findings of this study reaffirm the managerial advantages of calving early in a calving season for a *B. indicus-B. taurus* cow population. Bourdon and Brinks (1983) found that on average, a *B. taurus* cow’s subsequent calving date was delayed 0.11 d for each 1-d delay in the previous calving date. If this trend were to manifest in a cow’s reproductive timeline, at some point she would be biologically unable to recover after parturition and return to estrus before the end of the breeding season and would therefore fall out of production (Bourdon and Brinks 1983). In this study, a heifer that calved earlier in the season had a longer calving period between her first and second calves, although this did not appear to negatively impact her potential for longevity. Rather, this likely reflects the added time allowed for these early calving heifers to recover before rebreeding, aiding in their future success in achieving stayability at different ages (Diskin and Kenny 2014). Consequently, even though late-calving heifers rebreed following parturition, this timing may induce more stress and impede future reproduction.

*B. indicus-B. taurus* heifers that calve earlier in the season have increased opportunities to meet managerial benchmarks such as successive pregnancy and rebreed success and weaning a high proportion of her own body weight. If they were born in an early calving period, there is an even greater cumulative advantage for cow productivity. This study provides evidence that genetics can influence calving period of birth. This may be due to individual or breed differences in gestation length, age at puberty, or response to environmental stresses. Future studies using larger or designed datasets may be useful for parsing apart these interrelated effects. Combined, this suggests that cattle genetics can be optimized for a production system typical of Central Texas, where *B. indicus*-crossbred cattle are regularly managed to calve as heifers at 2 yrs of age for successive future calvings.
